# Optimizing Depression Classification Using Combined Datasets and Hyperparameter Tuning with Optuna

**DOI:** 10.3390/s25072083

**Published:** 2025-03-26

**Authors:** Ștefana Duță, Alina Elena Sultana

**Affiliations:** Applied Electronics and Information Engineering, National University of Science and Technology POLITEHNICA Bucharest (U.N.S.T.P.B.), 060042 Bucharest, Romania; stefana.duta@stud.fim.upb.ro

**Keywords:** EEGNet, depression classification, Optuna optimization, EEG signal processing, machine learning, hyperparameter tuning, clinical diagnostics, neural networks

## Abstract

**Highlights:**

**What are the main findings?**

- The study proposes an EEGNet model optimized with Optuna for depression state classification, using data from two public databases and an independent one, achieving 93.27% accuracy on the independent Proprietary dataset.
- The main contribution of this study lies in its approach to unifying EEG datasets collected from different sources, providing a method for real-world applications where data are more diverse and not confined to a single location.

**What is the implication of the main finding?**

- The results demonstrate the potential of hyperparameter optimization, offering a framework for handling real-world EEG data variability for depression detection. Unlike recent research, which primarily focuses on individual publicly available datasets, this work emphasizes dataset integration to enhance scalability. The objective was not solely to achieve high classification accuracy but to develop a framework that facilitates the unification of multiple datasets.
- The study proposes a standardized pipeline for preprocessing and classification, ensuring that the method remains applicable across different EEG datasets. By utilizing a lightweight neural network, the approach is designed to be easily integrated into portable systems, enhancing accessibility in real-world clinical settings.

**Abstract:**

This research focuses on the depression states classification of EEG signals using the EEGNet model optimized with Optuna. The purpose was to increase model performance by combining data from healthy and depressed subjects, which ensured model robustness across datasets. The methodology comprised the construction of a preprocessing pipeline, which included noise filtering, artifact removal, and signal segmentation. Additive extraction from time and frequency domains further captured important features of EEG signals. The model was developed on a merged dataset (DepressionRest and MDD vs. Control) and evaluated on an independent dataset, 93.27% (±0.0610) accuracy with a 34.16 KB int8 model, ideal for portable EEG diagnostics. These results are promising in terms of model performance and depression state-of-the-art classification accuracy. The results suggest that the hyperparameter-optimized Optuna model performs adequately to cope with the variability of real-world data. Furthermore, the model will need improvement before generalization to other datasets, such as the DepressionRest dataset, can be realized. The research identifies the advantages of EEGNet models and optimization using Optuna for clinical diagnostics, with remarkable performance for deployed real-world models. Future work includes the incorporation of the model into portable clinical systems while ensuring compatibility with current EEG devices, as well as the continuous improvement of model performance.

## 1. Introduction

Depression is a major global health challenge, and its prevalence has increased further in recent years due to the impact of the COVID-19 pandemic, as well as the current lifestyles of people, which are much more solitary and involve much less outdoor movement and therefore lower levels of vitamin D [1,2]. The increasing prevalence of depressive symptoms highlights the need for an objective early diagnostic method. Traditional methods, those based on subjective questionnaires, are prone to errors and delayed detection.

Electroencephalography (EEG) is now a common and reliable option for finding depression markers and making diagnosis and treatment checking more objective [3]. Recent EEG methods use deep learning to improve depression classification accuracy. For example, EEGDepressionNet [4] introduced a framework that mixes Self-Attention-based Gated DenseNet with a Chaos Owl Invasive Weed Search Optimization algorithm. This model extracts features from EEG signals using 3D-CNN, 1D-CNN, and spectral analysis, achieving 94% accuracy on the DepressionRest Dataset.

Another important study, EDT [5], created a deep learning model that extracts features from the frequency domain, as well as spatial and temporal aspects of EEG data, achieving an accuracy of 92.25%. EDT focuses on exploring the frequency domain feature extraction module and attention mechanism, which effectively identify patterns specific to depression. In a third study, DCST [6] proposed an attention network designed to use the spatial distribution characteristics of EEG data for depression recognition. The model achieved an accuracy of 89.8% with a leave-one-subject-out cross-validation method. DCST has both RegionalCalculationNet and GlobalCalculation-Net in its design, which pick up spatial and time features at both local and larger levels. But these studies also noted issues like small datasets and complicated models; they hinted that there needs to be further work performed on identifying more compact models, as well as testing on larger groups.

Previous research in the field [7,8] analyzed two approaches for classifying depression from EEG signals: the first involved the use of a simpler machine learning approach, such as the Multilayer Perceptron model that analyzes features extracted from the data; the second study used a basic version of the EEGNet structure applied directly to raw EEG signals. These methods have demonstrated the potential to differentiate between healthy and depressed individuals, although each had limitations in terms of noise interference, and feature selection. More recent efforts, like those of EEGDepressionNet, EDT, and DCST, have advanced feature extraction and model complexity, yet their focus on individual datasets limits their applicability across heterogeneous populations. This gap suggests an opportunity to integrate multiple datasets and refine model parameters to improve both accuracy and adaptability, particularly for clinical use where data variability is inevitable. Building on these foundations, the presented work aims to increase the accuracy of depression classification by pooling multiple datasets (DepressionRest along with MDD vs. Control) while tuning the hyperparameters of some networks using Optuna. The work introduces a series of improvements that aspire to improve the accuracy and generalization of depression detection, and these include noise filtering techniques to remove artifacts, feature selection to optimize classification, and hyperparameter optimization. The efficient processing of high-dimensional EEG signals is vital for real-time clinical applications. Compressed sensing techniques, such as the Adaptive Stepsize Forward-Backward Pursuit (ASFBP) method [9] or the optimal tensor truncation approach for multichannel EEG compression [10], offer a promising approach to enhance processing efficiency by reconstructing sparse signals using adaptive thresholds or by compressing multi-channel EEG data into a compact core tensor. While not implemented here due to our focus on raw EEG data and deep learning, CS could reduce sampling demands and computational load in future EEG frameworks, complementing our aim for lightweight, scalable depression diagnostics.

The central question guiding our research is as follows: Can combining multiple EEG datasets and optimizing deep learning model hyper-parameters with Optuna library [11] improve the generalizability of depression classification? Our aim is to develop a robust tool for clinical applications that can accurately diagnose depression using EEG data. To achieve this, we will develop a standardized preprocessing pipeline that includes noise filtering, artifact removal using independent component analysis (ICA), and feature selection. We will optimize deep learning models using Optuna, combine multiple datasets to increase training data diversity, compare optimized and non-optimized models, and validate results using statistical tests to confirm the significance of observed improvements across different datasets and configurations.

## 2. Materials and Methods

### 2.1. Database

The study uses 2 publicly available EEG datasets: Depression Rest (DR) database [12] and MDD vs. Control (MDD) database [13]. The DR dataset consists of EEG signals collected from 122 participants, selected based on their scores on the Baker Depression Scale survey. These participants were between 18 and 25 years of age and had no history of neurological disorders or substance use. Depression was classified according to Beck Depression Inventory (BDI) scores, ranging from 0 to 13 for non-depressed and 14 to 63 for depressed individuals. EEG data were recorded using 64 electrodes, sampled at a rate of 500 Hz.

The MDD dataset contains recordings from 30 healthy control subjects and 34 individuals diagnosed with major depressive disorder. Data for each subject were gathered under 3 different protocols: eyes closed, eyes open, and TASK, the latter involving visual stimuli to study evoked potentials. The signals were sampled at 256 Hz. For the purposes of this study, only the “eyes closed” data were considered from both databases to ensure EEG acquisitions that result in signals with fewer artifacts, allowing for a more reliable comparison between the two datasets.

**Figure 1:**
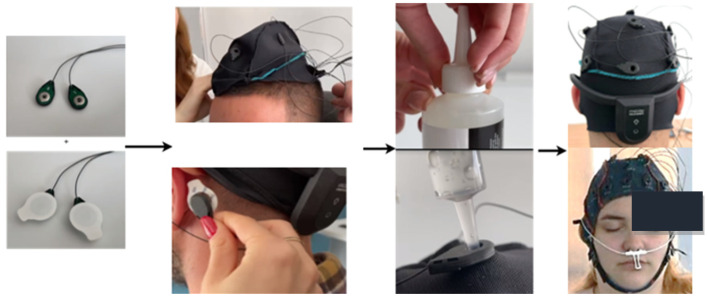
The image sequence shows the steps involved in setting up the EEG system: first, the ground and reference electrodes are shown. Next, the EEG cap is positioned on the head, followed by the attachment of the reference electrodes behind the ears. Conductive gel is applied to ensure proper contact, and the positioning of the electrodes is checked to ensure they are in the correct locations and maintain good contact with the scalp according to the 10–20 standard.

In addition to the open-source datasets, a self-collected dataset was also used for model testing, consisting of 2 subjects diagnosed with major depressive disorder and 4 clinically healthy subjects. The depressive subjects were diagnosed by neurologists at the time of signal acquisition. Additionally, all subjects completed a DASS-42 questionnaire [14] (Depression, Anxiety, and Stress Scales) before the signal acquisition. This is a 42-item self-report scale designed to measure negative emotional states of depression, anxiety, and stress. Its primary role in a clinical context is to clarify the nature of emotional disturbance as part of a broader clinical evaluation. The essential function of the DASS was to assess the severity of the main symptoms of depression, anxiety, and stress. The two depressive subjects scored highly on all three emotional states, while the others had low scores for anxiety and depression and moderate scores for stress. In the process of creating the custom dataset, the EEG Unicorn: The Brain Interface headset [15] was used. The EEG data from the brain activity recording at eight scalp positions (Fz, C3, Cz, C4, Pz, PO7, Oz, PO8) were sampled at 250 Hz per channel with a 24-bit resolution. The headset comes with pre-set positions for the electrodes, ensuring they conform to the 10–20 standard. Additionally, all subjects provided written consent in accordance with GDPR regulations. The process of acquiring the self-collected data is briefly presented in Figure 1 below.

### 2.2. Method

**Figure 2:**
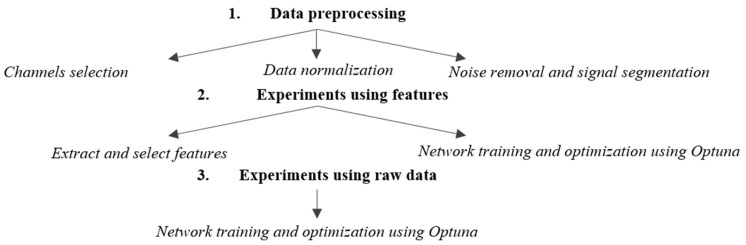
Paper workflow.

Figure 2 presents the EEG data processing workflow employed in this study to differentiate between patients with major depressive disorder and the control group.

The workflow consists of three main stages: data preprocessing, training/validation using extracted features, and training/validation using raw data. Both the feature-based method and the raw EEG data method include an automatic hyperparameter search step for optimizing the networks. The preprocessing stage, critical for ensuring data quality and consistency across datasets, involves three steps for both approaches. The first step is selecting common channels to ensure that the same cortical regions are analyzed across the two databases and a consistent input format is used. Next, the data are normalized to bring them to the same unit of measurement, and the final preprocessing step involves noise removal and signal segmentation to standardize the data length.

The next section focuses on feature extraction and training a model using the extracted information, as well as training a network derived from EEGNet using raw EEG data. Feature extraction involves identifying relevant attributes from the signals, such as dominant frequencies, frequency band power, temporal, and nonlinear characteristics. Feature selection then narrows down the most relevant attributes for classification, ensuring that only essential information is used in the subsequent analysis. The final step, network training, applies classification techniques to interpret the EEG data.

#### 2.2.1. Data Preprocessing

The preprocessing pipeline begins with channel selection, where eight common channels (Fz, C3, Cz, C4, Pz, PO7, Oz, PO8) are chosen from all datasets to ensure consistent cortical coverage and input format, aligning with the 10–20 international system. The next step is normalization. In this case, the EEG signals in µV from the DR database are converted to V.

Following a frequency analysis of the signals from the MDD database, a 50 Hz powerline noise was detected, which was removed using a 2nd-order Infinite Impulse Response (IIR) notch filter with a quality factor (Q) of 35, implemented in Python’s SciPy library (version 1.11.4). To isolate relevant signals and optimize the algorithm’s performance, a 5th-order Butterworth bandpass filter (1–40 Hz) is applied to the raw signal using a zero-phase filtering technique (SciPy’s filtfilt) for training models that use raw data. Frequencies outside this range are susceptible to noise and unwanted artifacts, which can negatively impact raw signal analysis. For the CNN network that involves feature analysis, the full frequency range (0.5–100 Hz) of the five bands (delta: 0.5–4 Hz, theta: 4–8 Hz, alpha: 8–13 Hz, beta: 13–30 Hz, gamma: 30–100 Hz) was preserved. For further signal cleaning, independent component analysis (ICA) was used. Among these, one of the first statistically independent components is often associated with eye blink artifacts, which exhibit a high-intensity region in the anterior frontal lobe. The FastICA algorithm was implemented in MNE-Python with default parameters (tolerance: 10^−4^, maximum iterations: 200). Eye blink artifacts can be eliminated without losing other useful information by identifying them via visual inspection and automated variance thresholding (>20% of total variance) and removing this specific component, and then reconstructing the signal using only the remaining independent components. ICA was applied to data with preserved rank, as preprocessing steps were performed in a way that did not reduce rank [16].

**Figure 3:**
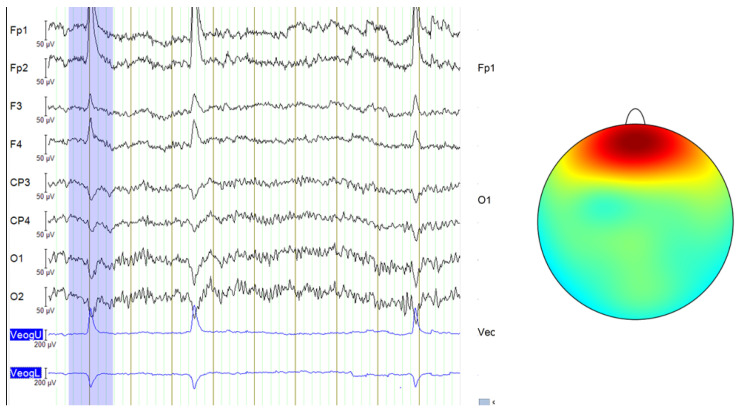
Blink artifacts in EEG data, with the last two channels representing an EOG signal that records eye movements (**left**) and the topographic map showing the typical scalp distribution of blinks (**right**—color gradients represent electrical activity across the scalp and red indicates high positive voltage, concentrated around the frontal electrodes) [10].

In this study, even though the acquisition protocol for all databases used required subjects to keep their eyes closed, some signals from the DR database still exhibited micro-blink artifacts or other eye movements similar to blinking, likely caused by muscle contractions. These artifacts are visible on the EEG recording and are characterized by very large amplitude spikes, as seen in Figure 3. The signals from the MDD database and the self-collected data followed a consistent eyes-closed acquisition protocol, leading to cleaner signals without such noise. Therefore, only some signals from the DR database required filtering using ICA to remove these micro-blink artifacts or muscle-induced eye movements.

Further, in the process of segmenting the signals into smaller fragments, the goal was to obtain an equal number of samples for subsequent analysis, specifically 1500 samples, regardless of the database’s sampling frequency. This was achieved by dividing the continuous EEG recordings into fixed-length time windows. Each window represented a smaller segment of the original signal while preserving its key characteristics. This approach ensured that all datasets contributed an equal number of training samples, preventing any imbalance between databases. This value was found to be optimal after several trials.

Lastly, the dataset was divided into a training and a testing set using an 80–20 split. For both databases, each subject was assigned to either the training or testing set based on their unique identifier. This approach ensured that data from the same subject did not appear in both sets simultaneously, preventing the risk of overfitting. To bolster model robustness, data augmentation was applied exclusively to the Proprietary dataset’s test set. Each original sample was augmented three times by adding Gaussian noise (mean = 0, standard deviation = 0.01) and randomly shifting the signal within ±10% of its length (150 samples), with values clipped to the original signal’s range. This augmented data evaluate the model’s ability to generalize to varied conditions, used only during testing for the raw data experiment.

#### 2.2.2. Experiments Using Features

The features used for the EEG signal analysis were categorized into three main types: time domain features, frequency domain features, and nonlinear features. Time domain features included the mean and the standard deviation. Additionally, kurtosis was used to distinguish the sharpness of the waveform, while asymmetry gave insights into the symmetry of the EEG signal. In the frequency domain, the power spectrum, obtained through Fast Fourier Transform (FFT) in NumPy, was analyzed. Power spectral ratios were also calculated to compare the power of various frequency bands within the signal. Lastly, nonlinear features, such as differential entropy, were employed to quantify the complexity of the brain’s electrical activity, offering a measure of signal uncertainty.

Since response time is important in clinical analysis, it is desirable to perform the analysis in as short a time as possible. Initially, a high number of features (157) is used, which requires a long time and high computing power. Therefore, relevant features for detecting depression are identified. Using SelectKBest (scikit-learn, k = 10), a univariate statistical method for feature selection, it was found that the DR set has the best features:

- Max Delta;
- Max Theta;
- Max Alpha_low;
- Max Gamma; and
- Max Gamma_low.

The MDD dataset’s best features include the following:

- Delta/Gamma;
- Delta/Gamma_low;
- Delta/Gamma_middle;
- Theta/Beta_high; and
- Theta/Gamma_middle.

No single set of features was found to be consistently optimal across both datasets. Instead of selecting a common subset, the 5 best features from each dataset were chosen, resulting in a set of 10 features that captured the most relevant information from both databases. This approach ensured that the classification model incorporated the strongest predictive features from both sources while maintaining computational efficiency.

In this study, an optimized neural network was developed using Optuna, a hyperparameter optimization framework that automates the search for optimal model configurations using Bayesian optimization with a Tree-structured Parzen Estimator (TPE) strategy. The TPE algorithm models the hyperparameter–performance relationship by estimating probability distributions of good versus poor configurations, iteratively sampling promising regions over 200 trials in a single optimization run. The search space included the number of convolutional layers (1–3), dense layers (1–3), convolutional filters (32, 64, 128), kernel size (3, 5), pooling size (2, 3), dense units (32–512), dropout rate (0.2–0.5), and learning rate (10^−5^ to 10^−1^, logarithmic scale). Each trial trained the model for 100 epochs with early stopping (patience: 10), evaluating accuracy on a validation set split from the 80–20 training data. Optimization terminated after 200 trials or a 24 h timeout (86,400 s), whichever occurred first, balancing exploration and computational cost. Table 1 exemplifies 10 trials, illustrating the range and outcomes (e.g., Trial 1: 69.65% accuracy with 128 filters). This single-run optimization, without repeated 200-trial cycles, aimed to maximize accuracy efficiently, leveraging TPE’s adaptive sampling to refine the CNN architecture. 

Finding the optimal hyperparameters is a meticulous and time-consuming task, as it requires extensive experimentation and fine-tuning to achieve the best balance between model complexity and performance. Manual tuning is often impractical due to the vast search space and the computational effort involved. Automating this process with Optuna significantly accelerates hyperparameter selection, allowing for an efficient and systematic exploration of the search space while ensuring better generalization of the model to unseen data. 

#### 2.2.3. Experiments Using Raw Data

To enhance the EEGNet model’s stability and performance, hyperparameter optimization was conducted using Optuna with a Bayesian TPE approach, where TPE iteratively refines the search by modeling high-performing hyperparameter distributions over 200 trials in a single optimization run. The search space comprised kernel length (16–128, step = 16), F1 filters (4–16), depth multiplier D (1–4), F2 filters (8–48), dropout rate (0.2–0.5), normalization rate (0.1–0.5), and learning rate (10^−5^ to 10^−2^). Each trial trained the model for 100 epochs with a batch size of 64, using 5-fold KFold cross-validation with shuffling to compute validation accuracy, and early stopping (patience = 4) based on validation loss to prevent overfitting. Optimization terminated after 200 trials or a 24 h timeout (86,400 s), ensuring sufficient exploration. The 5-fold cross-validation evaluated the model post-optimization, not during the 200-trial search, which occurred once on the combined training set. This strategy maximized validation accuracy, adapting EEGNet to dataset variability without repeating the full 200-trial optimization, relying on TPE’s pruning of underperforming trials to focus computational resources. GPU usage was monitored using NVIDIA-smi when available, with memory growth configured for efficiency. Training occurred on the combined dataset, with testing on combined, DR, and augmented Proprietary sets. Efficiency metrics were computed, including total trainable parameters, million floating-point operations (FLOPs) via TensorFlow’s profiler, inference time averaged over 100 GPU predictions (in milliseconds), and model sizes for float32 and int8 quantized versions (in KB) using TensorFlow Lite, with int8 quantization calibrated on 100 training samples. Quantized accuracy is assessed on 100 test samples post-quantization.

Statistical analyses were conducted to compare and validate the performance of the EEGNet models across different configurations and datasets. For each dataset—Combined, DR, and Proprietary (Augmented)—paired *t*-tests, implemented using SciPy’s ttest_rel function, were employed to evaluate the statistical significance of accuracy differences between the Baseline and Optimized EEGNet models. These tests were applied to the accuracy scores obtained from 5-fold cross-validation, where each fold yields an accuracy metric for both models on the respective test sets. The paired *t*-test assumes that the accuracy measurements are paired by fold, assessing whether the Optimized model’s enhancements (e.g., tuned hyperparameters) result in a statistically significant improvement over the Baseline model. The test returns a t-statistic and a *p*-value for each dataset, with a *p*-value threshold of 0.05 indicating significance.

To further explore the Optimized model’s consistency across datasets, a one-way Analysis of Variance (ANOVA), implemented via SciPy’s f_oneway function, was performed using the cross-validation accuracies from the Optimized model runs on the Combined, DR, and Proprietary (Augmented) datasets. This test assesses whether there are significant differences in mean accuracy among the three datasets, testing the null hypothesis that all dataset accuracies are equal. The ANOVA yields an F-statistic and a *p*-value, with a *p*-value less than 0.05 rejecting the null hypothesis and suggesting that at least one dataset’s performance differs significantly. When the ANOVA indicated significance (*p* < 0.05), a post hoc Tukey Honestly Significant Difference (HSD) test, executed using statsmodels.stats.multicomp.pairwise_tukeyhsd, was conducted to pinpoint which specific dataset pairs differ. This test constructs a DataFrame combining the accuracy scores from all folds across the three datasets, labeled by dataset name, and computes the mean difference, *p*-value adjusted for multiple comparisons, and confidence intervals at an alpha level of 0.05. The Tukey HSD results provide a detailed breakdown of pairwise comparisons, ensuring clarity on how dataset-specific factors (e.g., sampling frequency, augmentation) influence model performance.

Complementing these statistical tests, a computational efficiency report is generated to aggregate and analyze key performance metrics, with a focus on evaluating the models’ suitability for clinical portable applications, such as deployment on resource-constrained devices like the ESP32 microcontroller with 4 MB FLASH memory. This report consolidates efficiency metrics calculated during training and evaluation, including the total number of trainable parameters, million floating-point operations (FLOPs) derived from TensorFlow’s profiler, inference time averaged over 100 GPU predictions (measured in milliseconds), and model sizes in both float32 and int8 quantized formats (in kilobytes) obtained via TensorFlow Lite conversion. The float32 model size was computed directly from the TFLite-converted model, while the int8 quantized size leverages optimization with a representative dataset of 100 training samples to calibrate quantization parameters. Quantized accuracy was assessed on the first 100 test samples per dataset, processed through a TFLite interpreter with int8 input and output types, and compared against the float32 accuracy from the full test set evaluation. For each model (Baseline and Optimized) and dataset combination, the report details these metrics alongside mean test accuracy and standard deviation from cross-validation, as well as classification performance via precision, recall, and F1-scores.

This summary highlights the trade-offs between float32 and int8 formats for the Optimized model, calculating the percentage size reduction ((float32 size − int8 size)/float32 size × 100) and the accuracy drop (float32 accuracy − int8 accuracy), critical for assessing portability. The analysis for clinical applications evaluates whether the int8 model sizes fit within the ESP32’s memory constraints and if inference times support diagnostic use, noting that real-time applications requiring sub-10 ms latency may necessitate further optimization.

## 3. Results

A topographic map in EEG analysis is a visual representation of brain activity across different scalp regions over time. It provides an intuitive way to examine spatial signal distribution by mapping electrode positions onto a 2D projection of the head, typically using a heatmap-style color scale to indicate signal strength in microvolts (µV).

**Figure 4:**
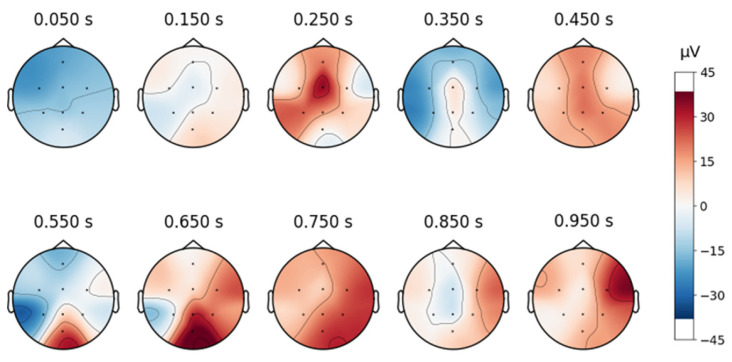
Topographic map of a signal in the database MDD.

In this study, time-based topographic maps were used to identify potential artifacts in the EEG signals. These maps are constructed by capturing the electrode positions at multiple time points and visualizing the intensity of electrical activity across the scalp. Areas with stronger signals appear in warmer colors, while weaker signals are shown in cooler tones. The primary goal of this analysis was to ensure that no strong noisy sequences were present, which could disrupt multiple channels, and to check for characteristic artifact patterns, such as those caused by teeth clenching or involuntary eye movements. As shown in Figure 4, the signals from the MDD database do not exhibit noticeable noise, particularly in the frontal lobe.

**Figure 5:**
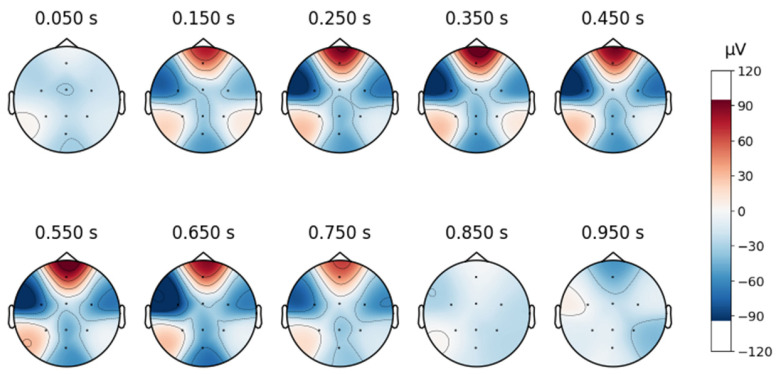
Topographic map of a signal in the database DR.

In contrast, although the DR database was collected under a closed-eye protocol, some signals still contain blinking artifacts, as illustrated in Figure 5.

To eliminate this noise, independent component analysis (ICA) was employed.

An eight-component representation was chosen, from which the component labeled “ICA000” was identified as corresponding to the blinking artifacts. This component was removed, and the signal was then reconstructed.

**Figure 6:**
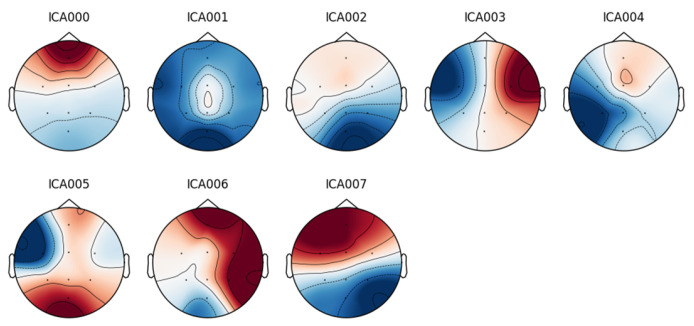
Independent component analysis of a DR database signal from a patient with blink artifacts.

**Figure 7:**
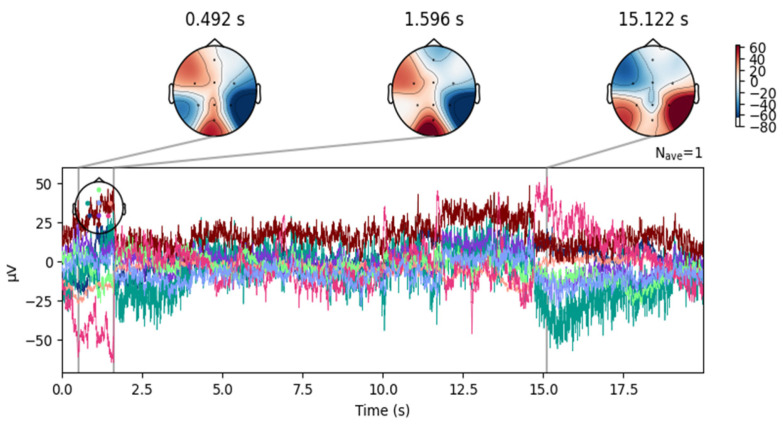
Topographical maps over time, associated with the waveform of an EEG signal from the DR dataset after removing the blinking artifacts.

Despite artifact removal techniques, some regions of the EEG signals remained noisy. As shown in Figure 6, the signal after removing the ICA component associated with blinking shows successful elimination of blinking artifacts in the frontal area. However, as shown in Figure 7, the signals still contain moving artifacts.

**Figure 8:**
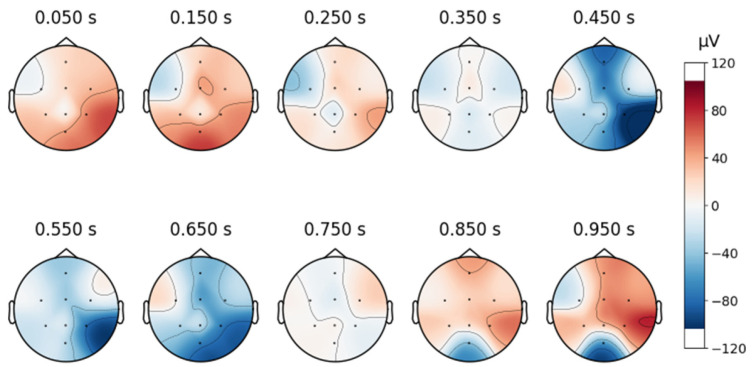
Topographic map of a signal in the custom database.

To continue analyzing the collected signal, topographic maps are created as shown below, in Figure 8, and it can be observed that the signals are free from artifacts.

### 3.1. Experiments Using Features

To obtain the highest possible results, the structure of a network was searched using Optuna. With the help of the library, the model that has the best accuracy is followed. Various parameters were adjusted during experiments, including the following:

- Number of convolutional layers: Between 1 and 3 layers.
- Number of dense layers: Between 1 and 3 layers.
- Number of convolutional filters: Choices of 32, 64, or 128 filters.
- Kernel size: Dimensions of 3 or 5.
- Pooling size: Dimensions of 2 or 3.
- Number of dense units: Between 32 and 512 units.
- Dropout rate: Between 0.2 and 0.5.
- Learning rate: Between 10^−5^ and 10^−1^ and on a logarithmic scale.

**Figure 9:**
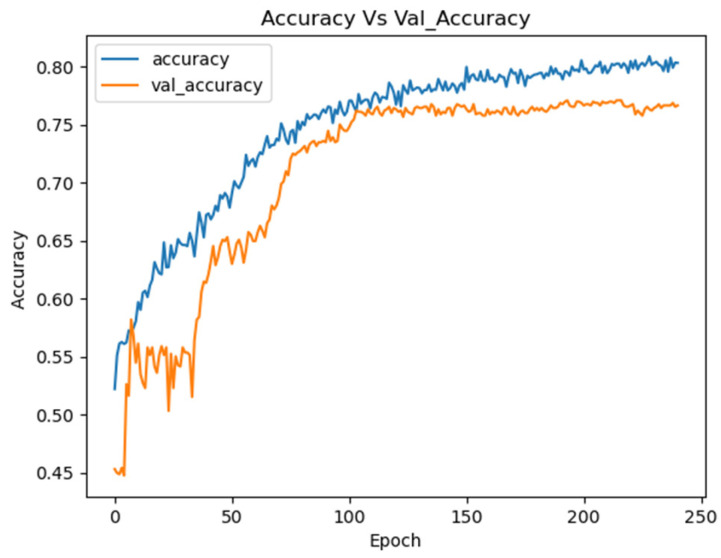
Training curves for the Optuna CNN on the combined datasets.

An example of an experiment with 10 trials using Optuna is presented in Table 1, detailing the hyperparameters and resulting accuracies. The Optimized CNN achieved an accuracy of 75.49%, with training and loss curves demonstrating stability and effective learning throughout the process (Figure 9). These results highlight the model’s ability to learn consistently, avoiding overfitting or underfitting.

Table 2 presents the confusion matrix for the CNN tested on the combined dataset. In this context, the labels used in the confusion matrix correspond to the classification of the subjects based on their health status. Label 0 represents the healthy class, while label 1 corresponds to the depressive class.

The model correctly classified 233 out of 302 instances labeled as 0 and 145 out of 208 instances labeled as 1, with false negatives and false positives of 63 and 69, respectively. The classification metrics are detailed in Table 3.

The CNN trained on the combined dataset was further evaluated on the Depression Rest dataset to assess its performance on noisier data. This test aimed to determine whether training on a combined dataset could enhance the model’s generalization to a dataset with lower quality or higher noise levels. The results are summarized in Table 4.

**Figure 10:**
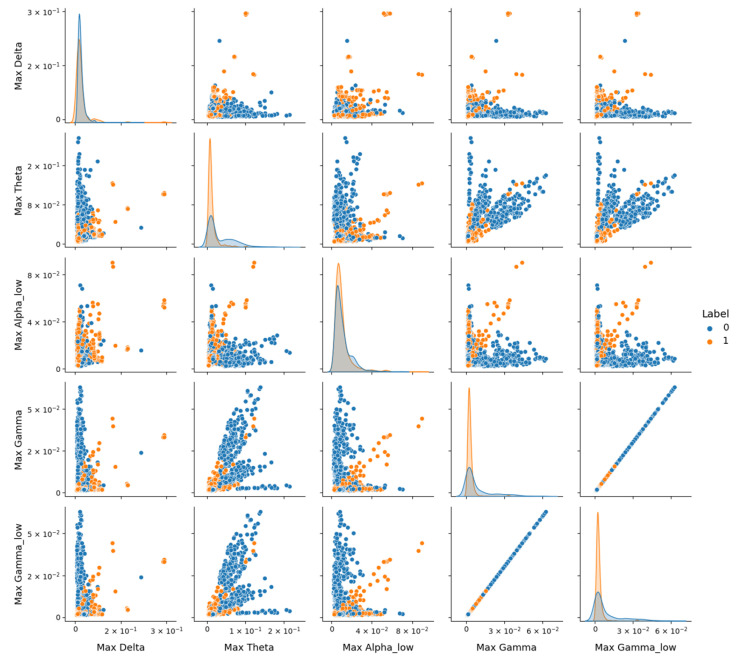
Representation of features from the DR dataset using the Seaborn Pairplot function. This visualization displays pairwise relationships between the most 5 discriminative features identified via SelectKBest, with scatter plots showing distributions for the healthy (Label 0) and depressive (Label 1) classes.

The study observed a notable decrease in model performance on the DR dataset, with the Optimized CNN achieving an F1-score of 0.56 for the depressive class (Class 1), as shown in Table 4. This contrasts with higher performance on the combined dataset (F1-score of 0.70, Table 3) and the Proprietary dataset (F1-score of 0.73, Table 5). To investigate this performance drop, pairplot visualizations using the version 0.12.2 of Seaborn library, as depicted in Figure 10, were employed to explore feature distributions in the DR dataset. Pairplot function creates a grid of scatter plots showing pairwise relationships between variables, with diagonal histograms illustrating individual feature distributions.

However, Figure 10 reveals significant overlap between the healthy (Class 0) and depressive (Class 1) classes across these features, despite their statistical relevance. Notably, depressive signals tend to exhibit higher amplitudes (e.g., in Max_Theta, Max_Gamma, and Max_Gamma_low), but the lack of clear separation suggests that noise or variability within the DR dataset may obscure class-specific patterns. The diagonal contains histograms of each feature’s distribution, highlighting the overlap between classes and the tendency of depressive signals to exhibit higher amplitudes in certain frequency bands.

**Figure 11:**
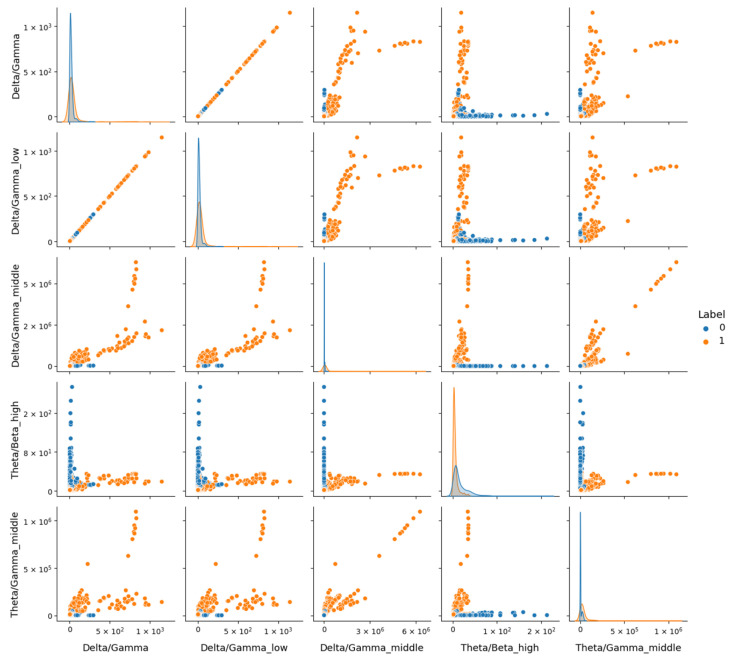
Representation of features from the MDD dataset using the Seaborn Pairplot function. This visualization displays pairwise relationships between the most 5 discriminative features identified, with plots showing distributions for the healthy (Label 0) and depressive (Label 1) classes.

In contrast, Figure 11 for the MDD vs. Control dataset shows a more pronounced distinction between classes, indicating that these features better capture depression-related differences in a less noisy context. Electrode configuration differences further complicate generalization. DR’s original 64-channel setup was reduced to eight common channels to align with other datasets, potentially discarding spatial information critical for depression markers, as seen in the topographic variability. The Proprietary dataset, natively collected with eight electrodes, and MDD, adjusted similarly, retained more relevant spatial data within this configuration, supporting higher performance.

These findings underscore the need for strategies to enhance robustness when dealing with datasets containing significant noise or variability.

To complete the analysis, testing was performed on the Proprietary dataset. The model achieved an accuracy of 72.87%, indicating consistent performance in classifying the depression state. However, for a more detailed evaluation, the results from Table 5 were also analyzed, showing balanced classification.

Although satisfactory results were obtained on the Proprietary dataset, the model could not generalize well on the Depression Rest database. As a result, alternative approaches are being explored. After analyzing the characteristics, no similar models were found, so the next approach uses raw EEG data.

### 3.2. Experiments Using Raw Data

The hyperparameter optimization for the EEGNet model was conducted using the Optuna library. An example of an experiment with 10 trials is presented in Table 6, detailing the hyperparameters and resulting accuracies.

Trial 5 achieved the best performance, with an accuracy of 0.7844, using a kernel size of 64, F1 (number of filters in the first convolutional block) of 16, D (depth multiplier) of 4, and F2 (number of filters in the second separable convolutional block) of 48. This suggests that a more complex and deeper network improved performance. Trial 9 had the lowest performance, with an accuracy of 0.6501, using a kernel size of 16, F1 of 4, D of 2, and F2 of 32. This indicates that the selected parameters were suboptimal, as the network was too small.

**Figure 12:**
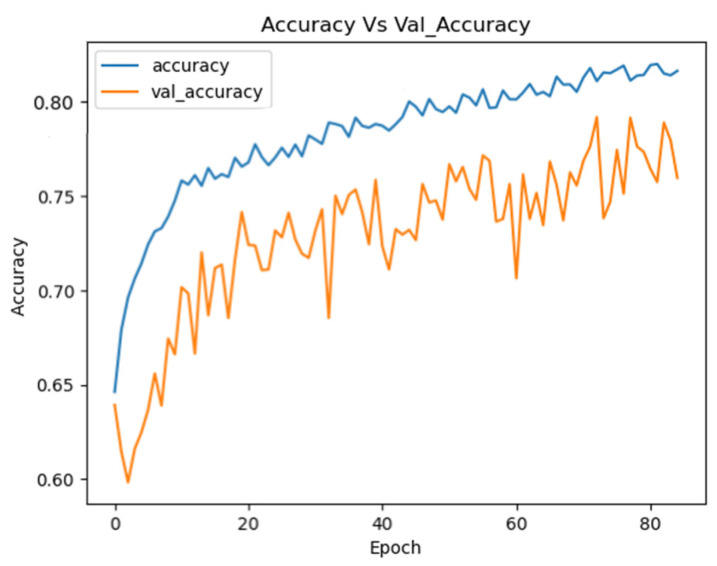
Training curves for the Optuna EEGNet on the combined datasets.

The Optimized EEGNet achieved a mean test accuracy of 80.85% (±0.0138) on the combined dataset, with float32 accuracy of 80.85% and int8 quantized accuracy of 77.40%. The inference time was 44.86 ms, and the int8 model size was 28.94 KB. Figure 12 illustrates the training curves for the EEGNet model optimized with Optuna on the combined dataset.

These curves demonstrate the stability of the model during training, showing continuous improvement in performance for both the training and validation sets.

The confusion matrix presented in Table 7 highlights the number of correct and incorrect predictions for each class.

This table shows that the Optimized EEGNet model correctly classified 1094 instances as label 0 and 1086 instances as label 1. The relatively low number of misclassifications (288 false negatives and 294 false positives) underscores the model’s solid performance in distinguishing between the two classes.

Table 8 provides a detailed evaluation of the Optimized EEGNet model’s performance, showing precision, recall, and F1-score for both classes.

The network was further evaluated on the Depression Rest (DR) dataset, achieving a mean test accuracy of 85.71% (±0.0142), float32 accuracy of 85.71%, and int8 quantized accuracy of 83.20%, with an inference time of 49.80 ms and an int8 model size of 28.94 KB. The results are summarized in Table 9.

The confusion matrix of the optimized model tested on proprietary dataset is presented in Table 10.

Testing on the Proprietary (Augmented) dataset yielded a mean test accuracy of 93.27% (±0.0610), float32 accuracy of 93.27%, and an impressive int8 quantized accuracy of 98.00%, with an inference time of 45.26 ms and an int8 model size of 28.94 KB. The results are detailed in Table 11.

The EEGNet model optimized with Optuna demonstrated excellent performance on the Proprietary dataset, confirming that the optimizations led to a robust and efficient model. The outstanding results indicate that the model not only trained well on the combined dataset but also managed to maintain its performance in the specific context of the Proprietary dataset. This suggests that the model is well-adapted for real-world applications, offering confidence in its practical use.

A summary of the results is presented in Table 12 and Table 13, detailing the computational efficiency and performance of the Baseline and Optimized EEGNet models across three datasets: Combined, Depression Rest (DR), and Proprietary. Table 12 describes the Baseline model, an EEGNet network without hyperparameter optimization, with a lightweight architecture of 2450 parameters and 6.94 MFLOPs. It achieves a consistent inference time of 44.84 ms on an NVIDIA Tesla P100 GPU, with model sizes of 13.92 KB (float32) and 9.59 KB (int8), yielding a 31.1% size reduction. The float accuracies were 72.83% (Combined), 71.88% (DR), and 82.90% (Proprietary), with quantized accuracies of 74.60%, 66.60%, and 91.40%, respectively. Mean test accuracies from 5-fold cross-validation were 72.83% (±0.0138), 71.88% (±0.0142), and 82.90% (±0.0610), reflecting moderate performance with higher variability on the smaller Proprietary dataset.

Table 13 presents the Optimized EEGNet model, enhanced via Optuna hyperparameter tuning, featuring 19,650 parameters and 60.13 MFLOPs. Inference times average 46.17 ms (44.86 ms Combined, 49.80 ms DR, 46.26 ms Proprietary), with model sizes of 80.86 KB (float32) and 34.16 KB (int8), achieving a 57.8% size reduction. Float accuracies improved to 80.85% (Combined), 85.71% (DR), and 93.27% (Proprietary), with quantized accuracies of 77.40%, 83.20%, and 98.00%, respectively, the latter possibly enhanced by quantization’s regularization effects. Mean test accuracies were 80.85% (±0.0138), 85.71% (±0.0142), and 93.27% (±0.0610), demonstrating superior performance and stability. These results support the study’s aim of unifying diverse EEG datasets for scalable, portable diagnostics, with the Optimized model balancing accuracy and efficiency for clinical use.

### 3.3. Computational Efficiency for Portable Applications

The int8 quantization reduced the Optimized model’s size by 57.8%, from an average float32 size of 80.86 KB to 34.16 KB across all datasets. This compact size is ideal for deployment on resource-constrained devices like the ESP32 microcontroller, which has 4 MB of FLASH memory. Inference times ranged from 44.86 ms (Combined) to 49.80 ms (DR) on a GPU, specifically the NVIDIA Tesla P100 provided by Kaggle Notebooks, a platform owned by Google LLC (Mountain View, CA, USA), which uses hardware from multiple vendors (e.g., NVIDIA (Santa Clara, CA, USA), Intel (Santa Clara, CA, USA)), where training and evaluation were conducted, suitable for many clinical applications, though sub-10 ms latency may require further optimization. Quantized accuracy occasionally exceeds float32 due to regularization effects during int8 calibration.

These results are summarized in Table 14.

### 3.4. Statistical Signifiance of Results—Baseline (Eegnet) vs. Optimized Model

The statistical tests provide a robust evaluation of the Optimized model’s performance relative to the Baseline and across datasets. Paired *t*-tests were as follows:

- Paired *t*-tests assessed whether the Optimized model’s accuracy significantly differs from the Baseline’s on a per-fold basis. The negative t-statistics indicate that the Optimized model’s mean accuracy exceeds the Baseline’s.

Combined dataset: t-stat = −4.59 and *p*-value = 0.0118—On the combined dataset, the Optimized model significantly outperforms the Baseline model (*p* < 0.05), with a moderate-to-large effect size. The negative t-statistic confirms higher accuracy for the Optimized model, likely due to hyperparameter tuning via Optuna enhancing generalization across the merged dataset.

DR dataset: t-stat = −21.3149, *p*-value = 0.0017 (highly significant)—highly significant improvement (*p* < 0.01) with an extremely large effect size (t ≈ −21.31). This suggests that the Optimized model markedly outstrips the Baseline on DR, possibly due to better handling of noise.

Proprietary (Augmented): t-stat = −2.2022, *p*-value = 0.0924 (not significant)—the difference is not statistically significant (*p* > 0.05), with a small-to-moderate effect size (t ≈ −2.20). Despite the Optimized model achieving higher accuracy, it does not reliably outperform the Baseline model. The *p*-value (0.0924) is close to significance, suggesting a trend toward improvement, but variability (high SD = ±0.0921) or small sample size (6 subjects, augmented to ~2×) may weaken statistical power.

- ANOVAF-stat = 19.5329, *p*-value = 0.0002 (significant differences across datasets).

The ANOVA tested differences in Optimized model accuracies across the three datasets, yielding a highly significant result (*p* < 0.001). The large F-statistic (19.53) confirms substantial variability in performance, rejecting the null hypothesis that accuracies are equal. This suggests dataset-specific factors (e.g., noise levels, electrode configurations, sample sizes) strongly influence the Optimized model’s effectiveness, necessitating post hoc analysis to pinpoint differences.

- Tukey HSD Post hoc Test.

The Tukey HSD test identified pairwise differences between datasets, adjusting for multiple comparisons (alpha = 0.05). Negative meandiff values indicate the second dataset has higher accuracy.

Combined vs. DR: meandiff = 0.0486, *p*-adj = 0.0274 (significant)—this modest gap may reflect DR’s cleaner signal post-ICA and better alignment with the training data (partly DR-derived), despite the combined dataset’s broader scope.

Combined vs. Proprietary: meandiff = −0.0978, *p*-adj = 0.0022 (significant)—this large difference underscores Proprietary’s advantage, likely its native 8-channel setup and lower noise

DR vs. Proprietary: meandiff = −0.1333, *p*-adj = 0.0002 (significant).

## 4. Discussion

This project discusses the issue of combining datasets from different sources to improve the classification of the level of depression using artificial intelligence. The findings argue that the joint training of several datasets improves the strength of the classifier while also enhancing its performance on sets of data that differ from the training set in real life. The Optimized EEGNet model achieved mean test accuracies of 80.85% (±0.0138) on the combined dataset, 85.71% (±0.0142) on Depression Rest (DR), and 93.27% (±0.0610) on the Proprietary dataset, with float32 accuracies of 80.85%, 85.71%, and 93.27%, and int8 quantized accuracies of 77.40%, 83.20%, and 98.00%, respectively. All these advances have important implications for the clinical use of these models, as they prove their feasibility for such scenarios where accuracy and repeatability are necessary. This can also be achieved in other biomedical research areas, which attests to its usefulness and importance.

Hyperparameter optimization via Optuna proved to be pivotal, enabling exploration of a vast parameter space—kernel length (16–64), filters (4–16, 8–48), dropout (0.2–0.5)—to refine the EEGNet architecture. Paired *t*-tests reveal significant improvements over the Baseline model, with t = −4.59 (*p* = 0.0118) on combined and t = −21.3149 (*p* = 0.0017) on DR datasets, indicating moderate-to-large and extremely large effect sizes, respectively. These gains likely stem from enhanced generalization and noise handling, as the Optimized model markedly outperforms the Baseline’s 72.83% (±0.0138) and 71.88% (±0.0142) on these datasets. However, on the Proprietary dataset, the improvement from 82.90% (±0.0610) to 93.27% yielded t = −2.2022 (*p* = 0.0924), a non-significant result despite a small-to-moderate effect size. This *p*-value, hovering near the 0.05 threshold, suggests a trend toward improvement, but its lack of significance warrants scrutiny. The Proprietary dataset’s small sample size—only six subjects (two depressed, four healthy), augmented three-fold via Gaussian noise and time shifts—may have contributed to this outcome, as limited data reduces statistical power and increases variability (±0.0610), the highest among datasets. Augmentation, while enhancing robustness testing, could introduce artificial patterns or overfitting to the augmented test set, particularly with the int8 model’s 98.00% accuracy, potentially inflating performance without corresponding statistical confidence in the float32 comparison. This contrasts with combined and DR datasets, where larger, more diverse samples (122 and 64 subjects, respectively) yield significant results. The Proprietary dataset’s clinical efficacy shines through its 98.00% int8 accuracy, validated across DR and combined testing, suggesting the model is finely tuned for depression diagnosis despite these statistical caveats. Collaboration with healthcare professionals during deployment could amplify these benefits, integrating new data to bolster sample size and clarify augmentation’s impact.

Comparative with other works that typically rely on individual datasets, this study offers a novel protocol for unifying and processing EEG data from multiple sources. The proposed method effectively addresses the challenge of dataset integration, ensuring the model is more robust and generalizable. For instance, EEGDepressionNet [4] achieves 94% accuracy on the Depression Rest dataset using a complex architecture combining 3D-CNN and 1D-CNN with spectral analysis, requiring approximately 1.2 million parameters and 2.5 billion FLOPs due to its Self-Attention-based Gated DenseNet structure. EDT [5] leverages multi-domain feature extraction (frequency, spatial, temporal) with an attention mechanism, employing around 800,000 parameters and 1.8 billion FLOPs, while DCST [6] uses a spatial attention network with roughly 600,000 parameters and 1.4 billion FLOPs, achieving 89.8% accuracy. In contrast, our Optimized EEGNet, with just 19,650 parameters and 60.13 MFLOPs, delivers a competitive 93.27% accuracy on the Proprietary dataset. This stark reduction in complexity—over 30 times fewer parameters and 40 times fewer FLOPs than EEGDepressionNet—highlights the proposed model’s simplicity and efficiency, making it more suitable for resource-constrained clinical applications.

Sharma et al. [17] (98.8%) similarly rely on extensive preprocessing and larger architectures, whereas our lightweight approach (34.16 KB int8) balances performance with practicality. DR’s lower accuracy reflects noise (micro-blinks, muscle artifacts) and electrode reduction, reducing spatial resolution critical for depression markers, as seen in pairplot overlaps versus MDD’s clearer separation. ANOVA confirms dataset-specific performance variations (F = 19.5329, *p* = 0.0002), rejecting equal accuracy across datasets, while Tukey HSD pinpoints Proprietary’s edge over combined (meandiff = −0.0978, *p*-adj = 0.0022) and DR (meandiff = −0.1333, *p*-adj = 0.0002), and DR’s modest superiority over combined (meandiff = 0.0486, *p*-adj = 0.0274). The Proprietary dataset’s native 8-channel setup and lower noise likely drive its advantage, contrasting with DR’s reduced 64-to-8 channels and noise challenges (micro-blinks, muscle artifacts) as seen in pairplot, overlaps versus MDD’s clearer separation.

The model’s lightweight design—int8 size of 34.16 KB (57.8% reduction from 80.86 KB float32)—and inference times of 46.17 ms across datasets on an NVIDIA Tesla P100 make it ideal for portable devices like the ESP32, enhancing clinical accessibility. The Baseline’s 72.83%, 71.88%, and 82.90% float32 accuracies rise to 74.60%, 66.60%, and 91.40% with int8, but the Optimized model’s 98.00% int8 on Proprietary outstrips these, though its >10 ms latency suggests optimization needs for real-time use (<10 ms). DR’s lower performance reflects noise and spatial resolution loss, a challenge not fully mitigated by ICA, unlike Proprietary’s cleaner native data.

The most important contribution of this work remains the fact that all models were trained on a very small number of electrodes, which is the configuration of most readily available EEG headsets. The fact that the Optimized EEGNet model performs well with a small dataset and no other requirement except for an EEG device makes the model capable and efficient for clinical testing. This enhances the applicability of the model in clinical settings, especially where resources are constrained. Ultimately, the goal of this study was not just to improve accuracy but to establish a scalable and efficient pipeline that unifies multiple EEG datasets, addressing the challenges of dataset variability and complex preprocessing. The results of this study confirm that the proposed method was successful in achieving these goals. Statistical tests (ANOVA: *p* = 0.0002) confirm dataset-specific performance, with Tukey HSD showing Proprietary’s superiority (*p*-adj = 0.0022 vs. Combined), likely due to native 8-electrode data versus DR’s reduced 64-to-8 channels, echoing noise challenges. The model’s compact size and 44.86–49.80 ms inference time suit portable devices like the ESP32, aligning with clinical needs for accessible diagnostics [3]. Int8 quantization (*p* = 0.0825, Proprietary) enhances efficiency without significant accuracy loss, contrasting with resource-heavy models [4,5]. By integrating several diverse datasets and optimizing hyperparameters, we not only enhanced the model’s generalization capabilities but also ensured its practicality for clinical environments where real-world data often varies. The proposed pipeline offers a robust solution for EEG data processing and classification, laying the foundation for future work in this field, particularly in expanding the model’s applicability across a wider range of clinical scenarios.

## 5. Conclusions

The research has presented advanced techniques for the diagnosis of depression utilizing EEG signals and machine learning models. There is an emphasis on collection and preprocessing methods that guarantee high-quality data.

The paper addressed the design and optimization of neural networks for EEG signal analysis, including the selection of models and initial hyperparameter values. Optuna was utilized for hyperparameter optimization, facilitating the efficient exploration of a large hyperparameter space. To discover the optimal parameter combinations, several experimental rounds were conducted. The methodology used in this study highlights the advantages and disadvantages of the approach, particularly regarding the classification of EEG features that distinguish between normal and depressive conditions.

Although the results are encouraging, several problems remain, particularly the need for additional data for testing and training, along with the challenges associated with processing EEG data. To enhance diagnostic precision, upcoming studies could explore the combination of various types of biometric data. Furthermore, in line with data protection laws, ethical issues regarding the utilization of EEG data, including confidentiality and the necessity for informed patient consent, need to be considered.

The enhanced EEGNet network will be incorporated into specialized devices for clinical applications in depression diagnosis in the future. A portable and adaptable processing module that can connect to different models of EEG headsets might be created to ensure compatibility with current models. Connecting to existing EEG headsets, developing suitable software for data collection and model operation, and presenting the findings in a manner that healthcare professionals can easily comprehend are all essential to implementing this strategy. This solution must be validated and supported in clinical settings to ensure its accuracy and reliability.

## Figures and Tables

**Table 1:** The 10 Optuna trials for the Optimized CNN on combined features.

Trial	Acc	Conv Layers	Dense Layers	Conv Filters	Kernel Size	Pool Size	Dense Units	Dropout Rate	Learning Rate
1 *	**0.6965**	**1**	**1**	**128**	**5**	**2**	**481**	**0.285**	**1.59 × 10^−5^**
2	0.4137	3	2	128	3	2	256	0.303	2.44 × 10^−4^
3	0.4104	2	3	64	5	2	283	0.390	6.20 × 10^−2^
4	0.5862	3	2	64	3	3	369	0.226	8.44 × 10^−2^
5	0.4148	2	3	64	3	2	492	0.408	1.30 × 10^−5^
6	0.4137	2	2	32	3	2	340	0.473	2.56 × 10^−4^
7	0.5552	1	1	128	5	3	156	0.312	1.05 × 10^−5^
8	0.4159	1	1	128	5	3	147	0.330	1.26 × 10^−5^
9	0.5873	1	1	128	5	3	151	0.341	1.42 × 10^−5^
10	0.5403	1	1	128	5	3	439	0.359	1.62 × 10^−4^

* Bold indicates the best trial based on the highest accuracy.

**Table 2:** Confusion matrix of the Optimized CNN on combined data.

	Real Label: 0	Real Label: 1
Predicted Label: 0	233	63
Predicted Label: 1	69	145

**Table 3:** Classification report for the Optimized CNN on combined data.

Label	Precision	Recall	F1-Score
Class 0	0.78	0.80	0.79
Class 1	0.71	0.69	0.70

**Table 4:** Classification report for the Optimized CNN tested on DR.

Label	Precision	Recall	F1-Score
Class 0	0.77	0.75	0.76
Class 1	0.53	0.57	0.56

**Table 5:** Classification Report for the Optimized CNN tested on Proprietary dataset.

Label	Precision	Recall	F1-Score
Class 0	0.76	0.81	0.75
Class 1	0.74	0.79	0.73

**Table 6:** 10 Optuna trials for the Optimized EEGNet on combined raw data.

Trial	Acc	Kernel	F1	D	F2	Dropout	Normalization	Learning Rate
1	0.7331	32	8	4	8	0.4428	0.1868	6.1600 × 10^−3^
2	0.7220	16	4	4	40	0.2528	0.2832	5.1000 × 10^−4^
3	0.6745	16	4	2	32	0.3124	0.1792	3.0700 × 10^−3^
4	0.6758	16	4	2	24	0.2467	0.3292	8.7510 × 10^−5^
5 *	**0.7844**	**64**	**16**	**4**	**48**	**0.4397**	**0.1900**	**1.2850 × 10^−3^**
6	0.7186	64	8	1	32	0.3183	0.4080	1.6600 × 10^−4^
7	0.6698	64	24	3	24	0.4247	0.4040	1.1900 × 10^−5^
8	0.7626	16	16	3	8	0.2866	0.1061	7.0500 × 10^−3^
9	0.6501	16	4	2	32	0.2516	0.4163	1.7240 × 10^−5^
10	0.7665	32	12	2	8	0.2405	0.1031	1.2700 × 10^−3^

* Bold indicates the best trial based on the highest accuracy.

**Table 7:** Confusion matrix of the Optimized EEGNet on combined data.

	Real Label: 0	Real Label: 1
Predicted Label: 0	1094	288
Predicted Label: 1	294	1086

**Table 8:** Classification report for the Optimized EEGNet tested on combined data.

Label	Precision	Recall	F1-Score	Metric	Value
Class 0	0.79	0.79	0.79	Mean Accuracy	80.85% (±0.0138)
Class 1	0.79	0.79	0.78	Float32 Accuracy	80.85%
				Int8 Accuracy	77.40%
				Inference Time	44.86 ms

**Table 9:** Confusion matrix of the Optimized EEGNet tested on DR dataset.

Label	Precision	Recall	F1-Score	Metric	Value
Class 0	0.80	0.77	0.81	Mean Accuracy	85.71% (±0.0142)
Class 1	0.78	0.79	0.78	Float32 Accuracy	85.71%
				Int8 Accuracy	83.20%
				Inference Time	49.80 ms

**Table 10:** Confusion matrix of the Optimized EEGNet tested on the Proprietary dataset.

	Real Label: 0	Real Label: 1
Predicted Label: 0	54	0
Predicted Label: 1	7	43

**Table 11:** Classification report for the Optimized EEGNet tested on the Proprietary dataset.

Label	Precision	Recall	F1-Score	Metric	Value
Class 0	0.89	1.00	0.94	Mean Accuracy	93.27% (±0.0610)
Class 1	1.00	0.86	0.92	Float32 Accuracy	93.27%
				Int8 Accuracy	98.00%
				Inference Time	46.26 ms

**Table 12:** Summary of the Baseline network results.

Baseline—Combined	Baseline—DR:	Baseline—Proprietary:
Parameters: 2450	Parameters: 2450	Parameters: 2450
FLOPs: 6.94 MFLOPs	FLOPs: 6.94 MFLOPs	FLOPs: 6.94 MFLOPs
Inference Time: 44.84 ms (GPU)	Inference Time: 44.84 ms (GPU)	Inference Time: 44.84 ms (GPU)
Model Size (Float32): 13.92 KB	Model Size (Float32): 13.92 KB	Model Size (Float32): 13.92 KB
Model Size (Int8): 9.59 KB	Model Size (Int8): 9.59 KB	Model Size (Int8): 9.59 KB
Float Accuracy: 0.7283	Float Accuracy: 0.7188	Float Accuracy: 0.8290
Quantized Accuracy: 0.7460	Quantized Accuracy: 0.6660	Quantized Accuracy: 0.9140
Size Reduction: 31.1%	Size Reduction: 31.1%	Size Reduction: 31.1%
Mean Test Accuracy: 0.7283 ± 0.0138	Mean Test Accuracy: 0.7188 ± 0.0142	Mean Test Accuracy: 0.8290 ± 0.0610

**Table 13:** Summary of the Optimized network results.

Optimized Model—Combined:	Optimized Model—DR:	Optimized Model—Proprietary:
Parameters: 19,650	Parameters: 19,650	Parameters: 19,650
FLOPs: 60.13 MFLOPs	FLOPs: 60.13 MFLOPs	FLOPs: 60.13 MFLOPs
Inference Time: 46.17 ms (GPU)	Inference Time: 46.17 ms	Inference Time: 46.17 ms
Model Size (Int8): 34.16 KB	Model Size (Int8): 34.16 KB	Model Size (Int8): 34.16 KB
Model Size (Float32): 80.86 KB	Model Size (Float32): 80.86 KB	Model Size (Float32): 80.86 KB
Float Accuracy: 0.8085	Float Accuracy: 0.8571	Float Accuracy: 0.9327
Quantized Accuracy: 0.7740	Quantized Accuracy: 0.8320	Quantized Accuracy: 0.9800
Size Reduction: 57.8%	Size Reduction: 57.8%	Size Reduction: 57.8%
Mean Test Accuracy: 0.8085 ± 0.0138	Mean Test Accuracy: 0.8571 ± 0.0142	Mean Test Accuracy: 0.9327 ± 0.0610

**Table 14:** Computational efficiency report.

Baseline Model (Average Across Datasets)	Optimized Model (Average Across Datasets)
Parameters: 2450	Parameters: 19,650
FLOPs: 6.94 MFLOPs	FLOPs: 60.13 MFLOPs
Inference Time: 44.84 ms	Inference Time: 46.17 ms
Float32 Size: 13.93 KB	Float32 Size: 80.86 KB
Int8 Size: 9.59 KB	Int8 Size: 34.16 KB
Float Accuracy: 0.7587	Float Accuracy: 0.8661
Quantized Accuracy: 0.7753	Quantized Accuracy: 0.8620
**Float vs. Int Comparison for Optimized Model**	
Average Float32 Size: 80.86 KB	
Average Int8 Size: 34.16 KB	
Size Reduction: 57.8%	
Average Float Accuracy: 0.8661	
Average Quantized Accuracy: 0.8620	
Accuracy Drop: 0.0041	

## Data Availability

The datasets used in this study are publicly available and were sourced from the following repositories: 1. Depression Rest (DR) Dataset: Available at Depression Rest—OneDrive. 2. MDD vs. Control Dataset: Available at MDD Patients and Healthy Controls EEG Data (New). 3. Proprietary Dataset: The self-collected dataset used for testing is not publicly available due to privacy and ethical restrictions. However, anonymized data may be made available upon reasonable request to the corresponding author, subject to ethical approval.

## Abbreviations

The following abbreviations are used in this manuscript:

EEG	Electroencephalography
MDD	Major depressive disorder
DR	Depression Rest Dataset
DASS-42	Depression, Anxiety, and Stress Scales (42-item)
ICA	Independent Component Analysis
CNN	Convolutional Neural Network
BDI	Beck Depression Inventory
DCNN	Deep Convolutional Neural Network
